# Supplementary material for: Cytotoxic and Antiproliferative Effects of Diarylheptanoids Isolated from Curcuma comosa Rhizomes on Leukaemic Cells
Source: Molecules. 2020 Nov 23;25(22):5476. doi: 10.3390/molecules25225476 (PMC7700379; doi:10.3390/molecules25225476)
Supplement: Supplementary file 1 [file molecules-25-05476-s001.pdf]

SUPPLEMENTARY DATA

Cytotoxic and Antiproliferative Effects of Diarylheptanoids Isolated from *Curcuma comosa* Rhizomes on Leukaemic Cells

Natsima Viriyaadhammaa [1], Aroonchai Saiai [2], Waranya Neimkhum [3], Wariya Nirachonkul [1], Wantida Chaiyana [4], Sawitree Chiampanichayakul [1,5], Singkome Tima [1,5,6], Toyonobu Usuki [7], Suwit Duangmano [1,5,*] and Songyot Anuchapreeda [1,5,6,*]

[1] Department of Medical Technology, Faculty of Associated Medical Sciences, Chiang Mai University, Chiang Mai 50200, Thailand; fai.natsima@gmail.com (N.V.); nirachonkul.w@gmail.com (W.N.); chiampanich@gmail.com (S.C.); singkome@gmail.com (S.T.)

[2] Department of Chemistry, Faculty of Science, Chiang Mai University, Chiang Mai 50200, Thailand; saiai_aroonchai@hotmail.com

[3] Department of Pharmaceutical Technology, Faculty of Pharmaceutical Sciences, Huachiew Chalermprakiet University, Samutprakarn 10250, Thailand; waranya.ne@gmail.com

[4] Department of Pharmaceutical Science, Faculty of Pharmacy, Chiang Mai University, Chiang Mai 50200, Thailand; aa_rx105@hotmail.com

[5] Cancer Research Unit of Associated Medical Sciences (AMS CRU), Faculty of Associated Medical Sciences, Chiang Mai University, Chiang Mai 50200, Thailand

[6] Center for Research and Development of Natural Products for Health, Chiang Mai University, Chiang Mai 50200, Thailand

[7] Department of Materials and Life Sciences, Faculty of Science and Technology, Sophia University, Tokyo 102-8554, Japan; t-usuki@sophia.ac.jp

* Correspondence: suwit.du@cmu.ac.th (S.D.); sanuchapreeda@gmail.com (S.A.); Tel.: +66-8875-13673 (S.D.); +66-5394-9237 (S.A.)

Academic Editors: Luisa Tesoriere and Alessandro Attanzio

Abstract: *Curcuma comosa* belongs to the Zingiberaceae family. In this study, two natural compounds were isolated from *C. comosa*, and their structures were determined using nuclear magnetic resonance. The isolated compounds were identified as 7-(3,4-dihydroxyphenyl)-5-hydroxy-1-phenyl-(1*E*)-1-heptene (**1**) and *trans*-1,7-diphenyl-5-hydroxy-1-heptene (**2**). Compound **1** showed the strongest cytotoxicity effect against HL-60 cells, while its antioxidant and anti-inflammatory properties were stronger than those of compound **2**. Compound **1** proved to be a potent antioxidant, compared to ascorbic acid. Neither compounds had any effect on red blood cell haemolysis. Furthermore, compound **1** significantly decreased Wilms' tumour 1 protein expression and cell proliferation in KG-1a cells. Both purified compounds decreased the WT1 protein levels in a time- and dose- dependent manner. Compound **1** suppressed cell cycle at the S phase. In conclusion, compound **1** has a promising chemotherapeutic potential against leukaemia.

Keywords: Zingiberaceae; *Curcuma comosa*; diarylheptanoids; cytotoxicity; antioxidant; anti-inflammatory; haemolysis; anticancer; Wilms' tumour 1

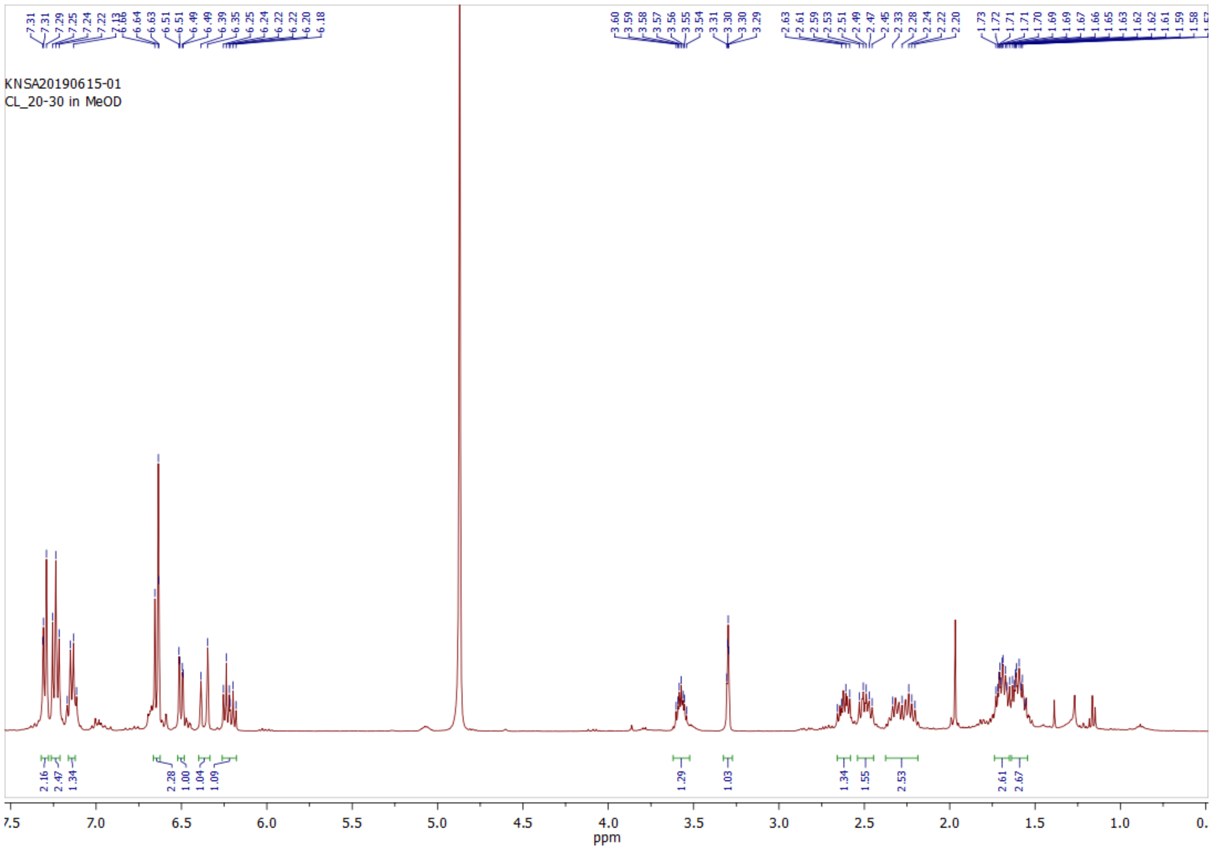

Figure S1. ^{1}H NMR spectrum of compound **1** in $CDCl_3$.

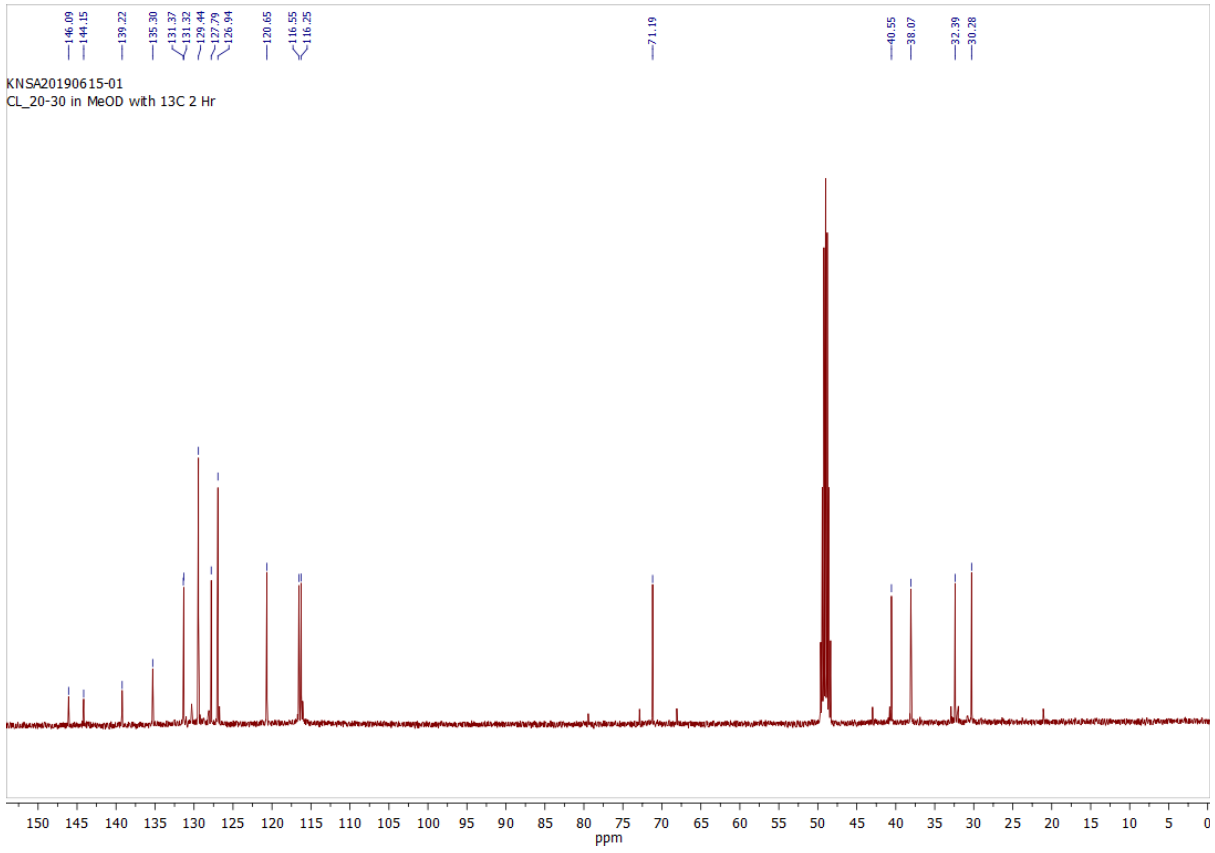

Figure S2. ^{13}C NMR spectrum of compound **1** in $CDCl_3$.

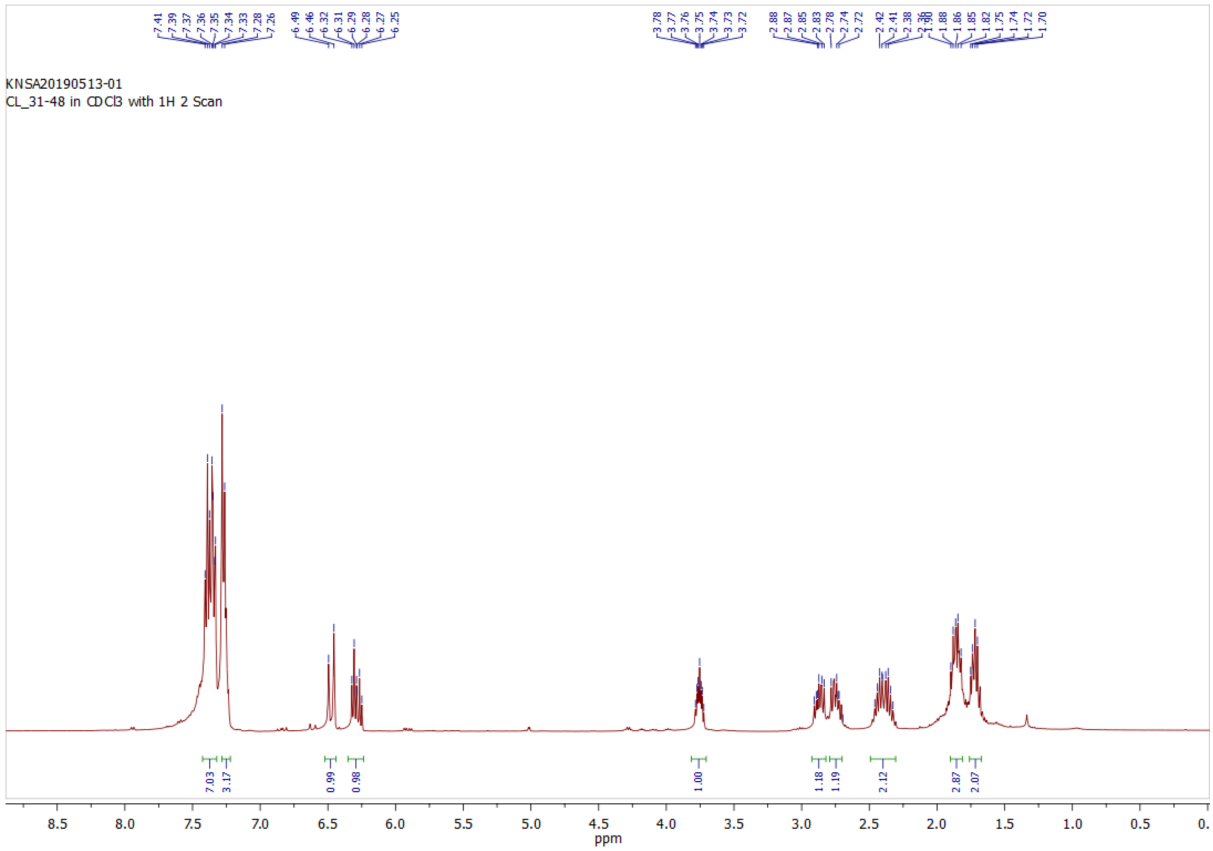

Figure S3. ^{1}H NMR spectrum of compound **2**.

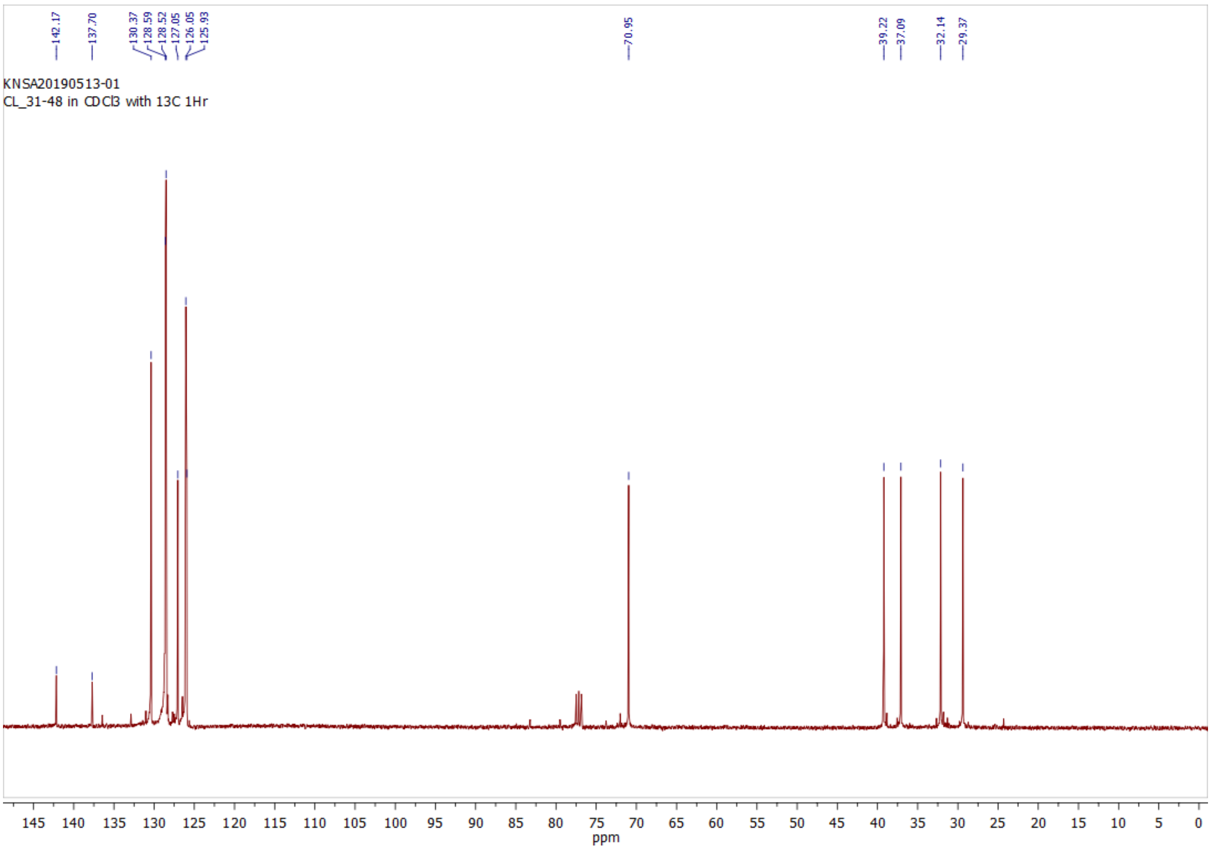

Figure S4. ^{13}C NMR spectrum of compound **2**.
